# Clinical Pharmacy in Radiopharmacy: A Cross-Sectional Survey of Practices Within the HUGOPharm Network

**DOI:** 10.3390/pharmacy14020056

**Published:** 2026-04-01

**Authors:** Evan Terrier, Laura Foucault-Fruchard, Nicolas Arlicot, Yann Venel, Mickaël Bourgeois, Serge Maia, Anne-Claire Dupont

**Affiliations:** 1 Radiopharmacy Department, Tours University Hospital, 37044 Tours, France; 2 Pharmacy Department, Tours University Hospital, 37044 Tours, France; 3 INSERM iBrain U1253, University of Tours, 37000 Tours, France; 4 Nantes-Angers Cancer Research Center CRCI2NA, University of Nantes, INSERM UMR1307, CNRS-ERL6075, 44000 Nantes, France; 5 Nuclear Medicine Department, Tours University Hospital, 37044 Tours, France; 6 Nuclear Medicine Department, Nantes University Hospital, 44000 Nantes, France

**Keywords:** radiopharmaceutical, clinical pharmacy, radiation safety, theranostic

## Abstract

Radiopharmacy is a specialized area of hospital pharmacy dedicated to the preparation and appropriate use of radiopharmaceuticals for diagnostic imaging and targeted therapies. While clinical pharmacy activities are well established in many hospital settings, their integration into radiopharmacy remains poorly documented and lacks standardization, particularly in the context of the rapid development of theranostic applications. This descriptive, cross-sectional study aimed to assess current clinical pharmacy practices in radiopharmacy across the HUGOPharm network. Between July and September 2025, an anonymous online questionnaire was distributed to radiopharmacy teams, collecting information on prescription analysis, biological monitoring, interdisciplinary collaboration, and other clinical pharmacy activities. Descriptive statistics were used to analyze responses. All participating centers reported verifying patient identity and key prescription parameters; however, substantial variability in practices was observed. Pharmacotherapeutic analysis was more frequently performed for therapeutic procedures (71%) than for diagnostic procedures (57%). Pre-procedure biological testing was common in therapeutic contexts (86%) but infrequent for diagnostic applications (29%). No center reported conducting structured medication reviews or pharmaceutical consultations. The implementation of radioligand therapies, such as [^177^Lu]-PSMA, was associated with enhanced safety checks and increased interdisciplinary collaboration. Overall, clinical pharmacy in radiopharmacy is developing but remains inconsistently implemented. Structured clinical pharmacy activities appear particularly relevant for theranostic procedures and may represent priorities for future practice development to support patient safety and integrated care.

## 1. Introduction

Radiopharmacy is a pharmacy specialty focused on the preparation and dispensing of radiopharmaceuticals (RPs), drugs containing radioactive isotopes used mainly in nuclear medicine. These compounds enable molecular imaging with Positron Emission Tomography (PET) or Single-Photon Emission Computed Tomography (SPECT) scanners, or deliver targeted radiation for therapeutic purposes, such as cancer treatment [1]. A RP is a compound typically composed of a radioisotope and a non-radioactive ligand. The radioisotope emits radiation—gamma rays, beta, or alpha particles—depending on its diagnostic or therapeutic use [2]. It is combined with a non-radioactive ligand that acts as a carrier, guiding the radioisotope to its target and ensuring it accumulates at the desired site. In some cases, such as iodine-131, the RP consists solely of the radioisotope, with biodistribution determined by its intrinsic properties [3].

By the 1950s, hospital pharmacists started to establish facilities to handle radioactive materials. The term “radiopharmacy” emerged as the practice became more specialized [4]. Hospital radiopharmacists played a key role in the production and dispensing of such preparations, as the short-lived radionuclides required fresh preparation and immediate administration to the patient [5]. The discipline combines radiochemistry, pharmacy practice, and health physics to ensure safe and effective clinical use. It shares tools and principles with pharmaceutics (e.g., aseptic techniques, GMP, clean rooms) but differs fundamentally by creating a new active compound through the combination of a carrier and a radioelement. Traditionally, the role of the radiopharmacist was limited to production, quality control, and dispensing of RPs. However, the discipline has evolved, and today, the radiopharmacist is also the healthcare professional legally responsible for managing and securing the RP supply chain, as well as ensuring its proper use [6]. This evolution aligns with the broader rise of clinical pharmacy, which emphasizes the pharmacist’s role in optimizing medication use and improving patient outcomes. Originating in the U.S. in the 1960s and later adopted in Europe, clinical pharmacy integrates cognitive, communicative, and collaborative skills into all stages of pharmacotherapy [7,8].

In radiopharmacy, this evolution has led to a progressive shift from purely technical activities toward more patient-centered responsibilities, including prescription analysis, assessment of potential drug–radiopharmaceutical interactions, and interdisciplinary collaboration. In this context, “clinical pharmacy in radiopharmacy” refers to activities extending beyond regulatory and technical validation, including pharmacotherapeutic analysis, identification of potential drug–radiopharmaceutical interactions, and contribution to medication management within a multidisciplinary framework.

Radiopharmacy is inherently complex due to the dual nature of radiopharmaceuticals, which combine a radioactive component with a non-radioactive ligand. In addition, dose prescription in radiopharmacy differs fundamentally from conventional pharmacotherapy, as administered activities are expressed in becquerels (Bq) at a given time rather than in mass units [9]. This specificity, together with radiation protection principles based on justification and optimization, places radiopharmacists in a key position to ensure accurate dose verification and the safe use of radiopharmaceuticals in both diagnostic and therapeutic settings [10].

This complexity is further illustrated by the distinction between diagnostic and therapeutic radiopharmaceuticals. Indeed, diagnostic RPs are usually administered in very small, safe doses, as their primary goal is to provide clear images without affecting the patient’s health. The term “radiotracer,” “tracer dose,” or “radiotracer dose” commonly refers to the use of a radiolabeled molecule in tiny (trace) amounts to study molecular processes [3]. According to the European Medicines Agency (EMA), a microdose is defined as less than 1/100th of the dose calculated to yield a pharmacological effect of the test substance. This calculation is based on primary pharmacodynamics (PD) data obtained by in vitro and in vivo studies. Typically, the maximum dose must be less than 100 μg or 30 nmol [11]. The use of such a low amount of the RP means that human body exposure is limited, so no therapeutic, toxic effects are expected. Thus, diagnostic RPs do not normally have any pharmacological effect, and their administration is not associated with major clinical side effects. Adverse reactions to diagnostic RPs are uncommon, usually transient and relatively minor in severity [12].

In contrast, therapeutic RPs may require higher amounts of the ligand to achieve the desired therapeutic effect, adding another layer of complexity to clinical management. The goal is to destroy or damage diseased cells while minimizing harm to surrounding healthy tissues. Therapeutic RPs are designed to have a more potent, localized effect, often over a longer period, to achieve their therapeutic purpose. Currently, there is intense interest in RP therapy within the framework of a theranostic paradigm that incorporates both diagnostic and therapeutic elements [13]. Ideally, this involves matched pairs of RPs: a diagnostic partner that provides information on disease extent and biological characteristics, and a therapeutic agent that delivers targeted radiation based on the diagnostic findings. Prominent examples include the use of [^68^Ga]-DOTATOC/TATE (EMA/FDA) for imaging and [^177^Lu]-DOTATATE for treatment of neuroendocrine tumors [14], as well as [^68^Ga]-PSMA PET imaging paired with [^177^Lu]-PSMA for prostate cancer therapy [15].

In this evolving landscape, RPs remain insufficiently integrated into broader frameworks for the proper use of medicines, despite being drugs associated with specific risks. With the advent of therapeutic RPs and the rapid development of nuclear medicine over the past 20 years, it is necessary to assess the current situation and define areas for improvement and prioritization in the proper use of these medications. The objective of this study was to provide a descriptive overview of current clinical pharmacy practices in radiopharmacy within a regional academic hospital network, and to identify heterogeneity and perspectives for future development in the context of increasing theranostic activity.

## 2. Materials and Methods

### 2.1. Study Design

This study was a descriptive, cross-sectional, observational survey conducted between July and September 2025. Its objective was to describe current clinical pharmacy practices in radiopharmacy within the French regional hospital pharmacy network HUGOPharm. HUGOPharm (Hôpitaux Universitaires du Grand Ouest) is a regional collaborative network bringing together pharmacy departments from university hospitals in western France. The network aims to promote collaboration, harmonization of professional practices, and sharing of expertise across hospital pharmacy activities. The present study focused specifically on radiopharmacy practices within this broader hospital pharmacy network. The study was reported in accordance with the CROSS (Consensus-Based Checklist for Reporting of Survey Studies) guideline [16].

### 2.2. Study Setting and Participants

The study was carried out within the HUGOPharm network, which brings together several French hospital radiopharmacies. All member institutions of the network at the time of the study were eligible to participate. The target population consisted of radiopharmacy teams involved in the validation, preparation, or pharmaceutical assessment of radiopharmaceutical prescriptions. Each participating institution was invited to provide one consolidated response representing its routine organizational and clinical pharmacy practices.

### 2.3. Questionnaire Development

A structured questionnaire was specifically developed for this study by a working group of hospital radiopharmacists with expertise in radiopharmacy and clinical pharmacy. Questionnaire development was based on a review of the literature and preliminary exploratory discussions conducted within the network to identify relevant clinical pharmacy activities and organizational practices. The questionnaire included both closed-ended questions (single- and multiple-choice) and open-ended questions allowing respondents to further detail local practices. The items were organized into thematic sections addressing:

(i) general organizational characteristics of radiopharmacy units;
(ii) regulatory and quality assurance activities related to radiopharmaceutical prescriptions (e.g., identity verification, prescription parameter checks, and dose/activity verification);
(iii) pharmaceutical assessment activities related to prescription validation, including the review of relevant biological parameters and pharmacotherapeutic evaluation of prescriptions with a specific focus on procedure-specific practices according to the type of nuclear medicine procedure.

Clinical pharmacy practices were analyzed according to the type of nuclear medicine procedure and classified into three main categories:

- Diagnostic procedures included primarily [^18^F]-FDG PET imaging, which represents the majority of PET activity, as well as common SPECT examinations such as myocardial perfusion imaging and endocrine investigations (e.g., thyroid imaging).
- Therapeutic procedures included the use of radiopharmaceuticals such as [^131^I] for thyroid diseases, [^177^Lu]-PSMA (Pluvicto^®^), and [^177^Lu]-DOTATATE (Lutathera^®^) for targeted radionuclide therapies.
- Cell radiolabeling procedures involved the radiolabeling of autologous cells (e.g., leukocytes or erythrocytes) for diagnostic purposes.

In this study, clinical pharmacy in radiopharmacy refers to pharmaceutical assessment activities extending beyond regulatory and technical validation, including the review of relevant biological parameters, pharmacotherapeutic evaluation, and screening for potential contraindications or drug–radiopharmaceutical interactions. These activities are conducted within the scope of pharmaceutical responsibility and do not include medical decision-making, such as therapeutic indication, treatment modification, or biological monitoring.

### 2.4. Data Collection

The questionnaire was distributed electronically using Google Forms to all eligible HUGOPharm centers. Participation was voluntary. Responses were collected anonymously, and no personal or patient-identifiable data were collected. Only one questionnaire per institution was retained for analysis. In case of incomplete responses, only fully completed questionnaires were included.

### 2.5. Data Analysis

Data were analyzed using descriptive statistics with Microsoft Excel (Microsoft Corp., Redmond, WA, USA). Categorical variables are expressed as frequencies and percentages. Analyses were performed at the institution level, with each participating center considered a single unit of analysis. Given the exploratory nature of the study and the limited number of participating centers, no inferential statistical analyses were performed.

### 2.6. Ethical and Regulatory Considerations

This study evaluated professional practices and did not involve patients, interventions, or the collection of personal health data. According to French regulations, it is classified as research not involving the human person. As the survey was fully anonymous and did not collect identifiable data, no formal declaration to the French data protection authority (CNIL) was required.

In France, radiopharmaceutical preparation and prescription validation fall within the regulatory responsibilities of hospital pharmacists. The practices described in this study reflect organizational implementations within the HUGOPharm network and may vary between institutions; they should therefore not be interpreted as standardized practices at the national level.

## 3. Results

### 3.1. Response Rate and Regulatory Compliance

**Figure 1:**
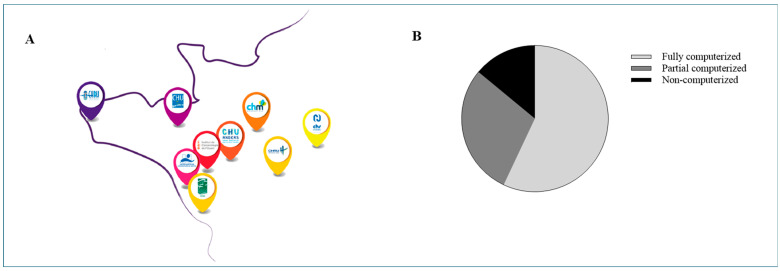
Results of the survey on clinical pharmacy practices in radiopharmacy within HUGO centers. (**A**) HUGOPharm centers with radiopharmacy. (**B**) Prescription system informatization.

**Figure 2:**
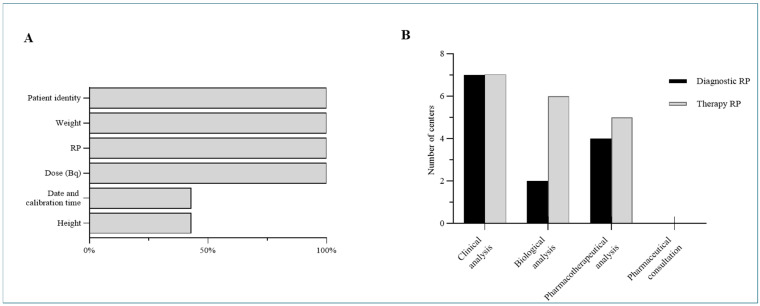
Results of the survey on clinical pharmacy practices in radiopharmacy within HUGO centers. (**A**) Regulatory analysis informatization. (**B**) Pharmaceutical analysis.

At the conclusion of the study period, 78% (7 out of 9) of the health facilities within the HUGOPharm radiopharmacy consortium completed the survey, including six university hospitals (Figure 1A). Among these centers, 57% (4 centers) used fully computerized prescription systems, 29% (2 centers) used hybrid systems, and 14% (1 center) lacked computerized prescription capabilities entirely, due to a lack of interface between imaging software and the RP preparation system (Figure 1B). Regarding regulatory compliance (Figure 2A), all centers (100%) systematically verified patient identity prior to RP preparation. However, verification of prescriber identity was only reported in the center using paper-based prescriptions, as this step is otherwise automated in digital systems.

### 3.2. Clinical Pharmacy Practices by Procedure Type

The survey revealed considerable heterogeneity in clinical radiopharmacy practices across participating centers (Table 1). In terms of pharmaceutical assessment of prescriptions, all surveyed centers (100%) reported systematically verifying the justification for both diagnostic and therapeutic nuclear medicine procedures, including assessments of patient weight, the prescribed RP, and activity (Bq) (Figure 2B). Despite this, only 43% of centers extended their verification to additional patient-specific parameters such as height or body mass index (BMI), as well as the calibration date and time of the RP. Notably, review of biological parameters (e.g., complete blood count (CBC), glomerular filtration rate (GFR)) is performed in only 29% of centers prior to diagnostic procedures to screen for potential contraindications, whereas this practice is substantially more common (86%) before therapeutic procedures (Figure 2B). Pharmacotherapeutic analysis—focused on identifying drug interactions and optimizing treatment regimens—is implemented by 57% of centers for diagnostic procedures and 71% for therapeutic ones. Notably, no center reported conducting formal pharmaceutical consultations with patients, regardless of procedure type. These results show variability in the implementation of clinical pharmacy activities across centers.

### 3.3. Diagnostic Procedures

Across diagnostic applications, clinical radiopharmacy involvement varied substantially depending on the procedure. For [^18^F]-FDG PET imaging, commonly used in oncology, radiopharmacy teams verified the appropriate delay between chemotherapy and the PET scan in 57% of centers. Control of patient fasting and glycemia was primarily managed by radio-operators (RDs) in 86% of centers, while administrative staff (AS) monitored the post-chirurgical delay in 57% of centers.

For [^68^Ga]-DOTATOC PET imaging, used to visualize somatostatin receptors in neuroendocrine tumors, radiopharmacists checked the delay after administration of somatostatin analogs in only 14% of centers. In myocardial perfusion imaging using ^99m^Tc-tetrofosmine, sestamibi, or ^201^Tl, which evaluate cardiac perfusion and viability, radiopharmacists were involved in verifying or coordinating the suspension of cardiology medications such as nitrates and beta-blockers in 57% of centers and calcium antagonists in 43%. Dietary restrictions (e.g., avoidance of bananas and chocolate) were enforced by RDs in all centers.

For renal imaging with ^99m^Tc-DMSA, a tracer that binds to the proximal renal tubules to assess cortical renal function, AS checked for appropriate delays after pyelonephritis in 43% of centers. In thyroid imaging with ^123^I, a radiotracer taken up by the sodium–iodide symporter in the thyroid gland, AS systematically ensured the suspension of antithyroid drugs and thyroid hormone replacement prior to imaging in 100% of centers.

Overall, these results highlight a variable but procedure-specific involvement of clinical radiopharmacy in diagnostic workflows, with variable integration for the management of hormonal or biological parameters.

### 3.4. Radiotherapeutic Procedures

For therapies involving ^177^Lu-DOTATATE and ^177^Lu-PSMA therapies—used respectively in neuroendocrine and prostate cancers—radiopharmacists verified renal function and CBC in 86% of centers. Additionally, physicians (MDs) evaluated renal, liver, and hematological parameters, including hemostasis, in 86% of centers. In the context of ^131^I therapy, administered for hyperthyroidism and differentiated thyroid cancers, the discontinuation of antithyroid drugs and thyroid hormone replacement was ensured in all centers (100%), coordinated either by MDs or AS. These results reflect a relatively high level of integration of clinical pharmacy in the preparation of therapeutic procedures, particularly for high-risk treatments.

### 3.5. Cell Radiolabelling

In procedures involving the radiolabelling of autologous cells, clinical pharmacy involvement was moderate and varied by cell type. For erythrocyte labelling with ^99m^Tc, used to assess blood pool and measure red cell volume, radiopharmacists verified the delay between any recent blood draw and the radiolabelling procedure in 57% of centers. In platelet labelling with ^111^In-aimed at assessing platelet survival and biodistribution, radiopharmacists ensured a minimum platelet count of 10,000/mm^3^ in 57% of centers. Similarly, leukocyte labelling with ^99m^Tc, a technique used to detect deep-seated infections or inflammation, a minimum leukocyte concentration of 3 G/L was verified by radiopharmacy teams in the same proportion of centers. These findings highlight a variability in pre-labeling safety checks across centers.

## 4. Discussion

This cross-sectional survey provides an overview of clinical pharmacy practices in radiopharmacy within the HUGOPharm network and reveals substantial heterogeneity between institutions. While regulatory compliance was consistently ensured across all participating centers, broader clinical pharmacy involvement varied considerably according to the type of nuclear medicine procedure.

A major finding of this study is the contrast between diagnostic and therapeutic practices. Although verification of patient identity and prescription parameters was systematic, only a minority of centers extended their analysis to additional patient-specific factors such as height or body mass index, and no center reported implementing structured pharmaceutical consultations. These results indicate that clinical pharmacy activities in radiopharmacy remain largely focused on regulatory checks rather than on comprehensive patient-centered interventions, particularly for diagnostic procedures.

In contrast, clinical pharmacy was more consistently integrated into therapeutic workflows. Pharmacotherapeutic analyses were performed more frequently before therapeutic procedures than diagnostic ones (71% vs. 57%), reflecting heightened vigilance in the context of radioligand therapies such as [^177^Lu]-DOTATATE and [^177^Lu]-PSMA. This difference can be interpreted within a risk-based perspective. Therapeutic radiopharmaceuticals are associated with clinically significant toxicities, particularly hematological and renal adverse effects, requiring careful patient selection, dose verification, and longitudinal monitoring [17,18]. In contrast, diagnostic radiopharmaceuticals are typically administered at tracer doses with limited pharmacological toxicity.

From this standpoint, the level of clinical pharmacy involvement appears to follow an implicit risk stratification logic, with more comprehensive pharmaceutical analysis implemented in higher-risk therapeutic settings. This finding supports the rationale for adapting the intensity of pharmaceutical interventions to the level of clinical risk associated with each type of radiopharmaceutical [19].

Clinical pharmacy involvement in diagnostic nuclear medicine was more variable across institutions. Although diagnostic radiopharmaceuticals are administered at tracer doses and are generally associated with limited pharmacological toxicity, inappropriate patient preparation or drug–radiopharmaceutical interactions may significantly affect tracer biodistribution and image quality. Such alterations may lead to false-negative or false-positive findings, with direct consequences for patient management. In this context, medication review prior to diagnostic procedures represents a relevant clinical pharmacy activity but remains inconsistently implemented, as previously reported [20,21,22].

Another notable finding is the absence of structured pharmaceutical consultations across all surveyed centers. This observation may be partly explained by the outpatient nature of most nuclear medicine procedures and the limited time patients spend in the department. However, the rapid expansion of theranostic approaches—particularly in prostate cancer—has progressively transformed nuclear medicine departments from predominantly imaging services into treatment-oriented care units. Therapeutic radiopharmaceutical administrations often require hospitalization and longitudinal follow-up, creating new opportunities and needs for clinical pharmacy involvement, including medication reviews and pharmaceutical interviews.

Taken together, these results suggest that clinical pharmacy practices in radiopharmacy are more developed for therapeutic applications than for diagnostic ones, but remain inconsistently implemented across institutions. The absence of standardized guidelines and clearly defined clinical roles likely contributes to this variability and represents a barrier to harmonized practice.

These findings should be interpreted within the context of the French healthcare system and its regulatory framework governing hospital pharmacy practice. In France, RP preparation and dispensing are strictly regulated, which may explain the consistent compliance observed across centers. However, the extent of clinical pharmacy involvement beyond regulatory requirements is not uniformly defined at the national level and may depend on local organizational structures. As such, the practices described in this study primarily reflect local initiatives within the HUGOPharm network rather than standardized national practices, and should not be directly extrapolated to other settings.

This study has several limitations. First, its cross-sectional design provides a snapshot of practices at a single point in time and does not capture their evolution. Second, the use of a single consolidated response per institution may not fully reflect intra-center variability, particularly in larger hospitals. In addition, the study was conducted within a limited number of institutions (seven respondents) belonging to a single inter-regional network, which may reflect specific local organizational practices. Therefore, the findings should be interpreted as representative of the HUGOPharm network rather than of all French or international settings. Furthermore, the questionnaire was not formally validated using psychometric methods, which may limit the reproducibility and robustness of the findings. Finally, as with all self-reported surveys, reporting bias cannot be excluded despite the anonymous nature of the data collection.

Beyond describing variability, this study provides several contributions to the understanding of clinical pharmacy practices in radiopharmacy. First, it identifies specific gaps in practice, particularly the limited implementation of medication reviews and the absence of structured pharmaceutical consultations, even in settings where radiopharmaceutical use is increasing. These findings highlight potential areas where patient safety and quality of care could be further strengthened. Second, the study supports a risk-based interpretation of clinical pharmacy involvement, showing that pharmaceutical activities are more consistently implemented in therapeutic settings associated with higher clinical risk. This perspective may help inform future frameworks for prioritizing clinical pharmacy interventions according to the level of risk associated with radiopharmaceutical use. Finally, by focusing on an inter-regional hospital network, this study provides original data on real-world practices in a field for which published data remain limited, thereby contributing to a better characterization of current clinical radiopharmacy practices.

## 5. Conclusions

Radiopharmacy has traditionally focused on the technical responsibilities of hospital pharmacists, particularly the production, quality control, and dispensing of radiopharmaceuticals (RPs). However, the increasing complexity of nuclear medicine, notably with the emergence of theranostic approaches, is progressively broadening the contribution of clinical pharmacy within multidisciplinary care. Radiopharmacists contribute to prescription analysis and regulatory verification of dose accuracy, especially given that RP dosing is expressed in becquerels (Bq) rather than mass.

This study highlights that clinical pharmacy practices in radiopharmacy remain heterogeneous and inconsistently implemented across institutions within the HUGOPharm network. These differences appear to follow a risk-based logic, with more consistent implementation of clinical pharmacy activities in therapeutic settings, where radiopharmaceuticals are associated with higher clinical risks, compared with diagnostic procedures involving tracer doses and lower pharmacological risk.

This variability may be explained by the absence of standardized national or international guidelines, as well as differences in local organizational practices [21,22]. Consequently, the findings should be interpreted as reflecting a regional organizational model and may not be fully generalizable to other settings.

Advanced clinical pharmacy activities, such as medication reviews and structured pharmaceutical consultations, remain inconsistently implemented but represent potential areas for development, particularly in theranostic procedures. In this context, clinical pharmacists may contribute to medication management and the identification of drug–radiopharmaceutical interactions as part of a multidisciplinary approach.

Future efforts aimed at developing harmonized clinical frameworks, supported by appropriate resources and institutional structures, may help strengthen patient-centered practices in clinical radiopharmacy. These developments should remain aligned with existing regulatory frameworks and clearly defined professional roles, ensuring that clinical pharmacy activities complement, rather than replace, medical decision-making and patient management.

## Figures and Tables

**Table 1:** Survey of Clinical Radiopharmacy and Nuclear Medicine Practices.

		Radiopharmaceutical	Physiopathological Mechanism	Main Indication	Clinical Radiopharmacy Practices Surveyed	Nuclear Medicine Practices (MD, RD, AS)
Diagnostic procedures	PET imaging	^18^F-FDG	Glucose metabolism	Oncology	-Delay verification between chemotherapy and PET exam 57% (4 centers)	-Blood glucose check (patient must be fasted) by RD (86%) -Delay verification between surgery and PET exam by AS (57%) -Screening diabetic patients (86)
		^68^Ga-DOTATOC	SSRT imaging	Neuroendocrine tumours	-Delay verification between somatulin analogue and PET exam 14% (1 center)	
	SPECT imaging	^99m^Tc-tetrofosmine/sestamibi or ^201^Tl	Passive diffusion Na^+^/K^+^ ATPase pump	Myocardial perfusion	-Suspension of cardiology drugs such as nitro-derivatives and beta-blockers 57% (4 centers) -Suspension of calcium antagonists 43% (3 centers)	-Avoidance of banana and chocolate consumption before examination by RD (100%)
		^99m^Tc-DMSA	Binds to proximal tubules at the renal cortex	Renal imaging		-Delay checking between pyelonephritis and TEMP exam by AS (43%)
		^123^I	Na^+^/I^−^	Thyroid imaging		-Discontinuation of antithyroid drugs and thyroid by AS (100%)
Radiotherapeutic procedures		^177^Lu-DOTATATE	SSRT IVR	Neuroendocrine tumours	-Renal function, CBC 86% (6 centers)	-Renal function, CBC, liver function, hemostasis parameters by MD (86%)
		^177^Lu-PSMA	PSMA IVR	Prostate cancer	-Renal function, CBC 86% (6 centers)	-Renal function, CBC, liver function, hemostasis parameters by MD (86%)
		^131^I	Na^+^/I^−^	Thyroid radiotherapy		-Suspension of antithyroid drugs and thyroid hormones by AS or MD (100%)
Cell radiolabelling	Erythrocyte	^99m^Tc-labeled erythrocyte		Measurement of globular volume	-Delay checking between bloodletting and exam 57% (4 centers)	
	Platelet	^111^In-labeled platelet		Determination of platelet survival and biodistribution	-Minimum 10,000 platelets required before radiolabelling 57% (4 centers)	
	Leukocyte	^99m^Tc-labeled leukocyte		Deep infections	-Minimum leukocyte count of 3G/L before radiolabelling 57% (4 centers)	

CBC, Complete blood count; IVR, Internal vectorized radiotherapy; MD, Medical Doctor, Physician; PSMA, Prostate specific antigen; RD, Radio operator; AS, Administrative staff; SSTR, Somatostatin Receptor Targeting; ^99m^Tc-DMSA, Technetium-99m dimercaptosuccinic acid; ^68^Ga-DOTATOC, Gallium-68 DOTA-D-Phe1-Tyr3-octreotide. Superscript numbers refer to the mass numbers of the radioactive isotopes (e.g., ^18^F, ^68^Ga), indicating the number of nucleons in the atom.

## Data Availability

The data presented in this study are available on request from the corresponding author due to privacy and ethical reasons.

## Abbreviations

The following abbreviations are used in this manuscript:

ALARA	As Low As Reasonably Achievable
Bq	Becquerel
CNP-MN	National Professional Council of Nuclear Medicine
CT	Chemotherapy
DRLs	Diagnostic Reference Levels
EANM	European Association of Nuclear Medicine
EMA	European Medicines Agency
ESCP	European Society of Clinical Pharmacy
FDG	Fluorodeoxyglucose
GMP	Good Manufacturing Process
HUGO	Hôpitaux Universitaires du Grand-Ouest
ICRP	International Commission on Radiological Protection
PD	Pharmacodynamics
PET	Positron Emission Tomography
QC	Quality Control
RP	Radiopharmaceutical
SG	Serum Glucose
SPECT	Single-Photon Emission Computed Tomography
